# Prevalence, healthcare costs and management of non-communicable diseases in people living with human immunodeficiency virus: A scoping review

**DOI:** 10.4102/phcfm.v12i1.2474

**Published:** 2020-10-19

**Authors:** Laston Gonah, Indres Moodley, Khumbulani Hlongwana

**Affiliations:** 1 Health Outcomes Research Unit, Discipline of Public Health Medicine, School of Nursing and Public Health, College of Health Sciences, University of KwaZulu-Natal, Durban, South Africa

**Keywords:** HIV/AIDS, co-morbidity, multi-morbidity, non-communicable diseases, hypertension, diabetes mellitus, prevalence, healthcare costs

## Abstract

**Background:**

Coexistence of human immunodeficiency virus (HIV) and non-communicable diseases (NCDs) is an important public health issue of increasing concern. However, the prevalence, healthcare costs and management protocols for NCDs in people living with HIV (PLHIV) remain unclear in most settings.

**Aim:**

To scope evidence on prevalence, healthcare costs and disease management protocols associated with NCDs in PLHIV from studies published before July 2019.

**Methods:**

Electronic databases were searched for published articles, and reference lists were checked for relevant studies. Key terms included were HIV/AIDS, co-morbidity or multi-morbidity, NCDs, healthcare costs, treatment protocols, diabetes mellitus, hypertension in various combinations.

**Results:**

A total of 152 records were assessed, and thereafter 25 studies were included in the final review after all the elimination. Twelve of the 25 studies mostly reported prevalence of NCDs in PLHIV, 4 reported impact of HIV–NCD co-morbidity on healthcare costs and 1 reported management protocols and capacity of antiretroviral therapy (ART) sites to manage HIV–NCD co-morbidity.

**Conclusions:**

Results showed higher prevalence rates of diabetes mellitus and hypertension in PLHIV compared with HIV-negative people. However, there was inconsistency in NCD prevalence data from studies conducted in sub-Saharan African (SSA) countries, and limited research evidence on capacity of ART sites to manage NCDs in PLHIV. Low prevalence rates of NCDs reported in SSA countries could be an indication of limited capacity to screen for NCDs because of the influence of health system and/or patient-level factors. Most studies were generally limited to cross-sectional studies, with very few interventional, longitudinal studies.

## Introduction

Unprecedented worldwide improvements in access to antiretroviral therapy (ART) have resulted in viral suppression and markedly increased survival for people living with human immunodeficiency virus (PLHIV). According to the Joint United Nations Programme on HIV/AIDS (UNAIDS), the number of PLHIV globally increased from an estimated 34 million in 2010 to 37.9 million people in 2018, with a concomitant 15% increase (from 47% to 62%) in the proportion of PLHIV accessing ART.^1,2^ As a result of increased access to ART since 2010, a 33% decline in acquired immune deficiency syndrome (AIDS)-related mortality globally was recorded by the end of 2018, demonstrating an increased survival in PLHIV as a result of ART. These statistics point towards a general increase in the survival of PLHIV as a result of ART.

Whilst ART has markedly increased the survival of PLHIV, the coexistence of HIV and non-communicable diseases (NCDs) has become an important public health concern. Evidence suggests that PLHIV are faced with relatively high rates of NCDs because of the following three main reasons: (1) from the HIV itself, (2) from the effects of ART and then finally and (3) from the increased risk associated with ageing.^1,3^ Undiagnosed, untreated or undertreated NCDs in PLHIV are likely to negate the positive gains already achieved in controlling HIV. However, the extent and magnitude of HIV-NCD co-morbidity, as well as the healthcare costs and disease management protocols in PLHIV, is generally not known.

We conducted a global scoping review to gain an understanding of the extent of the prevalence of NCDs in PLHIV as well as management protocols for HIV-NCD(s) co-morbidity and the healthcare costs of managing NCDs in PLHIV. The review only focussed on NCDs of global priority, that is cardiovascular diseases (CVDs), cancers, diabetes and chronic respiratory diseases.

## Methods

**FIGURE 1:**
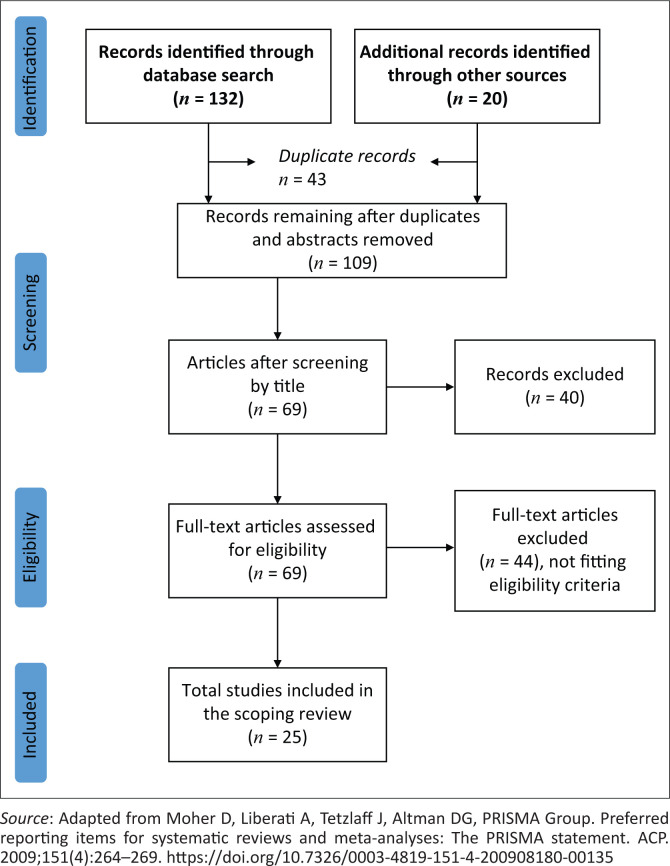
Preferred Reporting Items for Systematic Reviews and Meta-Analyses flow diagram summarising the search process and the selection results.

The scoping review was guided by the Preferred Reporting Items for Systematic Reviews and Meta-Analyses (PRISMA) statement, which provides a set of items guiding reporting in systematic reviews and meta-analyses.^4^ In Figure 1, the PRISMA clearly shows the stages of the scoping review.

### Search strategy

We conducted a scoping review of relevant studies from the following electronic databases: Google Scholar, Embase, PubMed, PubMed Central and African Journals Online.

The review study used a modified Population/Patient, Intervention/variable, Comparison, Outcome, Time and Setting/context (PICOTS) framework^5^ for formulation of study question and search syntax (Table 1).

**TABLE 1:** Modified Population/Patient, Intervention/variable, Comparison, Outcome, Time and Setting/context framework in search syntax formulation.

Population/patient	People living with HIV (PLHIV)
**I**ntervention/variables	Co-morbid HIV–NCDs, Integrated HIV–NCD management protocols
**C**omparison	PLHIV without NCDs, Vertical HIV and NCD care systems
**O**utcome	Prevalence, healthcare costs, NCD management protocols
**T**ime	Studies conducted before July 2019
**S**etting/context	Worldwide (low- to middle-income countries [LMICs], high-income countries [HICs])

*Source*: Samson D, Schoelles KM. Developing the Topic and Structuring Systematic Reviews of Medical Tests: Utility of PICOTS, Analytic Frameworks, Decision Trees, and Other Frameworks. In: Chang SM, Matchar DB, Smetana GW, et al., editors. Methods Guide for Medical Test Reviews [Internet]. Rockville (MD): Agency for Healthcare Research and Quality (US); 2012 Jun. Chapter 2. Available from: https://www.ncbi.nlm.nih.gov/sites/books/NBK98235/

Search terms used included (HIV or AIDS) AND (comorbidity or multi-morbidity) AND (non-communicable diseases), including cancers, cardiovascular diseases, diabetes mellitus, chronic respiratory diseases, prevalence in various combinations. Other search terms used were ‘HIV or AIDS’ AND ‘comorbidity or multi-morbidity’ AND ‘healthcare costs’ AND ‘treatment protocols’, AND ‘disease management protocols’. Reference lists of studies relating to HIV-NCD comorbidity were also checked to identify additional relevant literature.

### Inclusion criteria

Studies written in English language and published before July 2019 were included. All studies should have focussed on prevalence and/or incidence of NCDs in PLHIV, measuring healthcare costs of managing NCDs in PLHIV, or management protocols for NCDs in PLHIV.

Review articles, opinions, supplement letters, abstracts and viewpoints were excluded. Again, studies focussing on patient populations other than PLHIV were excluded.

### Methodological appraisal

The identified studies were assessed for quality by using an adapted 6-item quality assessment tool for systematic reviews of observational studies.^6^ The appraisal criteria include (1) representativeness of the sampling method used, (2) whether the sample size was statistically determined or whether it gave the study adequate power, (3) whether the eligibility criteria were clearly stated, (4) whether the outcome measures (NCD, healthcare costs) were objectively ascertained, (5) whether the outcome measures were clearly determined or assessed and (6) whether strategies to control for potential confounding were employed (e.g. stratification, matching, use of controls) in study design or data analysis. Each item was scored 1 point for a ‘Yes’ and 0 point for a ‘No’, giving a minimum possible total score of 0 and a maximum possible score of 6 (Table 2). Studies rated as poor were excluded from the final analysis.

**TABLE 2:** Scoring method for review articles.

Grading	5 or 6 out of 6	3 or 4 out of 6	0, 1 or 2 out of 6
Risk of bias	Low	Medium	High
Study quality	Good	Satisfactory	Poor
Number of articles identified (*n* = 65)	18 (27.7%)	7 (10.8%)	40 (61.5%)

*Source*: Adapted from Wong WCW, Cheung CSK, Hart GJ. Development of a quality assessment tool for systematic reviews of observational studies (QATSO) of HIV prevalence in men having sex with men and associated risk behaviours. Emerg Themes Epidemiol. 2008;5(1):23. https://doi.org/10.1186/1742-7622-5-23

### Review matrix

Summary of outcomes under study from each article was organised and presented in a Microsoft Excel (Version 2010) spreadsheet, coming up with a review matrix. Descriptive data analysis for quantitative data was performed as appropriate, whilst thematic analysis of qualitative data (consisting of statements on themes related to outcomes of interests) was performed in Nvivo (Version 12 ®).

### Ethical consideration

Biomedical Research Ethics Committee- University of KwaZulu-Natal BE086/19.

## Results

### Description of study articles selected

Twenty-five relevant studies meeting the inclusion criteria were identified. Most of the studies (17) included were conducted in Africa, specifically sub-Saharan Africa (SSA). The remaining ones were in Asia (2), Europe and America (6). The majority of the studies (20) were published between 2016 and 2018. Most of the studies (15) used non-experimental cross-sectional design, wherein two of them used HIV-negative comparison groups. The remaining 10 studies used modelling (2), retrospective cohort (4), intervention (3) and controlled time series quasi-experimental (1) designs. The majority of the studies (12) were primarily aimed at assessing the prevalence of NCDs and the associated risk factors or determinants, whilst the remaining were aimed at assessing the feasibility of integrated NCD–HIV management and outcomes (9), impact of co-morbidities (including hypertension and diabetes mellitus) on costs, utilities and health-related quality of life (HRQL) in PLHIV (4) and the capacity of HIV treatment facilities to provide care for people with NCDs (1).

### Magnitude of non-communicable diseases in people living with human immunodeficiency virus and the associated risk factors and/or determinants

#### Cardiovascular diseases

Hypertension was amongst the most common cardiovascular disease identified and one of the most evaluated NCDs in PLHIV (Table 3). Three studies used HIV-negative controls to compare the prevalence of hypertension in HIV-positive people with that of HIV negative people,^7,8,9^ and none of the studies compared people with HIV–NCD co-morbidity (as cases) with PLHIV without NCDs (as controls). The prevalence of hypertension amongst PLHIV was more than 10% in all but one study and was significantly higher in PLHIV than in HIV-negative controls. Studies conducted outside of Africa (with larger sample sizes), in Cambodia, Brazil, Italy and United States of America,^7,10,11^ found higher rates of hypertension in PLHIV than did the studies conducted from Africa. Again, there were marked variations in the prevalence of hypertension in the Malawian and Zimbabwean studies, with rates ranging 10.8% – 44.0% and 1.0% – 8.3%, respectively.

**TABLE 3:** Prevalence of common cardiovascular diseases.

Author, Year	Country	Main outcome variable	Prevalence		Sample size	Study design
			Cases (PLHIV)	Controls (HIV^−ve^)		
Chhoun et al.,^11^ 2017	Cambodia	Hypertension	15.1%	-	510	Cross-sectional
Magodoro et al.,^12^ 2016	Zimbabwe	Hypertension	10.2%	-	1033	Cross-sectional
Mutede et al.,^13^ 2015	Zimbabwe	Hypertension	30.0%	-	393	Cross-sectional
Rücker et al.,^8^ 2018	Malawi	Hypertension	10.8% – 44.7%	6.1% – 42.9%	735	Cross-sectional
Smit et al.,^10^ 2017	Italy United States of America	Moderate CVD (hypertension and dyslipidaemia)	60% in 2015 and 85% in 2035 (Italy) 61% in 2015 and 84% in 2035 (United States of America)	-	7469 3748	Modelling
Smit et al.,^9^ 2018	Zimbabwe	Hypertension	25.0%	5.6%	Population	Modelling
Galant et al.,^14^ 2017	United States of America	Essential hypertension	31.4% – 76.2%	-	64 398	Retrospective
Lorenc et al.,^15^ 2014	United Kingdom	Hypertension	5.0%	-	285	Retrospective
Ruzicka et al.,^16^ 2018	Japan	Hypertension	18.2%	-	1145	Retrospective
Serrao et al.,^17^ 2018	Portugal	Arterial hypertension	39.7%	-	401	Cross-sectional
Maciel et al.,^7^ 2018	Brazil	Hypertension	62.0%*	69.7%*	416	Cross-sectional

* *p =* 0.121 (statistically insignificant difference).

#### Diabetes mellitus

Diabetes mellitus was an often-mentioned metabolic disease and NCD in PLHIV, and the prevalence was demonstrated to be higher in PLHIV than in HIV-negative controls (Table 4). The prevalence of diabetes was found to be higher in studies conducted outside Africa compared with those conducted in Africa. Malawi and Zimbabwe showed marked variations in the prevalence of diabetes mellitus, ranging from 5% to 13% and 1% to 8.3%, respectively.

**TABLE 4:** Prevalence of diabetes mellitus in people living with human immunodeficiency virus.

Author, year	Country	Main outcome variable	Prevalence		Sample size	Study design
			Cases (PLHIV)	Controls (HIV^−ve^)		
Chhoun et al.,^11^ 2017	Cambodia	Diabetes mellitus	8.8%	-	510	Cross-sectional
Machingura et al.,^18^ 2017	Zimbabwe	Diabetes mellitus	8.3%	-	60	Cross-sectional
Maciel et al.,^7^ 2018	Brazil	Diabetes mellitus	22.6%	28.4%	416	Cross-sectional
Magodoro et al.,^12^ 2016	Zimbabwe	Type 2 diabetes mellitus	2.1%	-	1033	Cross-sectional
Rücker et al.,^8^ 2018	Malawi	Diabetes mellitus	5.0% – 13.2%	1.7% – 4.2%	735	Cross-sectional
Smit et al.,^10^ 2017	Italy United States of America	Diabetes mellitus	9.0% in 2015 and 27.0% in 2035 12.0% in 2015 and 23.0% in 2035	-	7469 3748	Modelling
Smit et al.,^9^ 2018	Zimbabwe	Diabetes mellitus	1.0%	0.4%	Population-based	Modelling
Lorenc et al.,^15^ 2014	U.K	Diabetes mellitus	11.2%	-	285	Retrospective
Galant et al.,^14^ 2017	United States of America	Diabetes mellitus	11% – 37%	-	64 398	Retrospective
Ruzicka et al.,^16^ 2018	Japan	Diabetes mellitus	26.8	-	1445	Retrospective
Serrao et al.,^17^ 2018	Portugal	Diabetes mellitus	13.5%	-	401	Cross-sectional

#### Cancers

Five of the studies selected assessed the prevalence of cancers in PLHIV, and two of them compared the prevalence of various forms of cancers in PLHIV with the prevalence in HIV-negative people.^7,9^ However, the major forms of cancer (cervical, breast and prostate) were very low (less than 1%), as reported by a study by Smit et al.^9^

#### Chronic respiratory diseases

Two studies, conducted in Zimbabwe, identified asthma as a problem in PLHIV (Table 5) and one study demonstrated that the risk of asthma is more prevalent in PLHIV than in HIV-negative controls. Studies conducted elsewhere did not identify chronic respiratory diseases as a common problem in PLHIV.

**TABLE 5:** Prevalence of asthma in people living with human immunodeficiency virus.

Author, year	Country	Main outcome variable	Prevalence		Sample size	Study design
			Cases (PLHIV)	Controls (HIV^−ve^)		
Magodoro et al.,^12^ 2016	Zimbabwe	Asthma	4.3%	-	1.033	Cross-sectional
Smit et al.,^9^ 2018	Zimbabwe	Asthma	1.2%	0.4%	Population based	Cross-sectional

### Common risk factors and determinants of non-communicable diseases in people living with human immunodeficiency virus

About six studies assessed the risk factors and/or determinants associated with NCD co-morbidity in PLHIV. Across all studies, patient’s age, HIV-positive status, duration of HIV infection and time on ART were often mentioned as factors positively associated with HIV–NCD co-morbidity^7,8,12,13^ (Table 6). Other studies also mentioned lifestyle factors (unhealthy diets, physical inactivity, harmful use of alcohol and smoking) as risk factors positively associated with HIV–NCD co-morbidities in PLHIV.^11,13^ In two studies, other social determinants of health such as gender and employment status were also found to be associated with NCDs in PLHIV, where being female and being unemployed were found to be independently associated with increased risk of development of NCDs^11,12^; however, the effect of these on access to treatment were not assessed.

**TABLE 6:** Common risk factors and determinants of non-communicable diseases in people living with human immunodeficiency virus.

Author, year	Title	Country	NCD(s)	Associated risk factor(s) and/or determinants	*p* AOR/POR
Chhoun et al.,^11^ 2017	High prevalence of non-communicable diseases and associated risk factors amongst adults living with HIV	Cambodia	Hypertension Diabetes Hypercholesterolemia	• Use of lard for cooking	0.01
				• Urban living	0.00
				• Less fruit consumption	0.02
				• Unemployment	0.03
				• Underweight	0.00
				• ≤ US$ monthly income	0.01
Maciel et al.,^7^ 2018	Co-morbidity is more common and occurs earlier in persons living with HIV than in HIV-uninfected matched controls.	Brazil	NCD multi-morbidity (CVDs, diabetes, neoplasia, hypertension)	• HIV positive Status	0.00
				• Duration of HIV infection	0.03
				• Time on ART	0.02
				• Age (≥ 50 years)	0.00
Magodoro et al.,^12^ 2016	A cross‑sectional, facility-based study of co-morbid non-communicable diseases amongst adults living with HIV infection	Zimbabwe	HIV–NCD co-morbidity/multi-morbidity (hypertension, asthma, cancer, type 2 diabetes)	• Age: 45 – ≤ 55 years	AOR 2.25
				• ˃ 55 years	AOR 5.42
				• Female gender	AOR 2.12
Mutede et al.,^13^ 2015	Prevalence and factors associated with hypertension amongst ART patients	Zimbabwe	Hypertension	• ART duration ˃ 2 years	POR 2.23
				• Waist-to-hip ratio ˃ 0.85 (women)	POR 3.45
				• BMI ˃ 25	POR 2.18
				• Smoking	POR 5.06
				• Sedentary recreation	POR 3.16
				• High salt intake	POR 2.67
Rücker et al.,^8^ 2018	High rates of hypertension, diabetes, elevated low-density lipoprotein cholesterol, and cardiovascular disease risk factors in HIV-infected patients	Malawi	Hypertension, diabetes mellitus, cardiovascular disease	• HIV infection, ART duration	-
Serrao et al.,^17^ 2018	Non-AIDS-related co-morbidities in people living with HIV-1 aged 50 years and older: The AGING POSITIVE study	Portugal	Non-AIDS-related co-morbidities (NARC), (including hypertension, diabetes mellitus, non-AIDS-related malignancies)	• Age	0.00
				• Duration of HIV-1 infection	0.00

AOR, adjusted odds ratio; POR, prevalence odds ratio.

### Impact of co-morbidities (including diabetes mellitus and cardiovascular diseases) on costs, utilities and health-related quality of life (HRQL) amongst people living with human immunodeficiency virus

Four studies reviewed assessed the impact of co-morbidities on costs, utilities and/or HRQL in PLHIV, from various perspectives. In two of the studies, NCDs (mostly mild CVDs including hypertension and diabetes mellitus) were not found to have a significant impact on healthcare costs (health system costs) in PLHIV, compared with the costs for HIV care alone.^18,19^ However, in a study conducted in Colombia to assess the impact of co-morbidities (including diabetes mellitus and CVDs) on costs, utilities and HRQL amongst PLHIV, a significant proportion (72%) of PLHIV experienced co-morbidities, and more than 50% experienced two or more co-morbidities, with the results showing a statistically significant impact of co-morbidities on utilities amongst patients with two or more co-morbidities, and some impact on HRQL of life.^19^ A study conducted by Shade et al.^18^ in Uganda found that integrating HIV care with care for hypertension and diabetes can improve the care for people with co-morbid HIV–NCDs. The study found that integrating NCD care (hypertension and diabetes mellitus) with the existing HIV care programme would only need an additional 4% – 5% of the total HIV care costs including expanding the care (diagnosis and treatment) to HIV-negative people with NCDs (4% marginal increase over HIV care cost in PLHIV and 5% marginal increase over HIV care cost in HIV-negative people). Cost categories considered were fixed costs (equipment), recurring goods costs (medications) and services (laboratory tests).

Smit et al.^10^ conducted a modelling study and performed projections of NCDs and healthcare costs amongst HIV-positive people in Italy and the United States of America. The NCD treatment costs, being driven to a greater extent by mild CVD (dyslipidaemia and hypertension) and diabetes in both countries, as a proportion of the total direct HIV costs, were estimated at 11.1% for Italy and 39.6% for the United States of America in 2015.

None of the studies attempted to assess the effect of healthcare costs (healthcare provider costs and patient costs) on health outcomes in people with HIV–NCD co-morbidity in SSA. None of the studies reviewed attempted to assess the effect of social determinants of health (income, employment status, level of education, sex, distance to health facility and HIV-NCD co-morbidity status) on healthcare costs, access to medication, hospitalisations, productivity and health outcome (relapse and mortality).

### Management of HIV-NCD comorbidity

Nine studies reviewed assessed the feasibility of integrated HIV–NCD screening programme. Four of these studies conducted in Swaziland, Kenya, Uganda and South Africa assessed the feasibility and yield of integrating diabetes and hypertension screening into HIV testing and care programme. Results of the studies consistently found that integrating diabetes and hypertension screening into HIV care programmes is feasible and provides potential for efficient resource utilisation compared with vertical and stand-alone screening programmes. However, the known yield of integrating HIV–NCD screening was found to be low, as demonstrated by three of the studies, which found a net NCD diagnosis yield of less than 5%. The other study conducted in Uganda found a high burden of undiagnosed HIV, diabetes mellitus and hypertension through the implementation of a community-based HIV–NCD screening programme.^20^

One study in South Africa assessed the effectiveness of the integrated chronic diseases management (ICDM) in controlling patients’ CD4 count and blood pressure, using the existing data from ICDM pilot facilities compared with the data from the control facilities. The study found the programme to be feasible, but with a relatively low yield of the ICDM where the likelihood of controlling CD4 and blood pressure was 6% and 1%, respectively.

Across all studies, integrating NCD screening into HIV screening and care programmes was found to be feasible, with potential for efficient resource utilisation compared with stand-alone programmes. Patient attendance or ensuring follow-up diagnosis in health centres was found to be a challenge, although determinants or reasons to this were not explored.

### Capacity of antiretroviral therapy centres to manage non-communicable diseases

Capacity of ART centres to manage NCDs is an important issue considering co-morbid HIV–NCDs becoming increasingly common in PLHIV. However, only one study conducted in Malawi assessed the capacity of ART centres to provide care for hypertension and diabetes mellitus.^21^ The study found that specialised human resources for HIV–NCD care, drug supply and screening were generally more limited to larger hospitals than health centres. Research evidence could not be found, focussing on the capacity of health centres and associated challenges to screen for NCDs in SSA.

## Discussion

The study made important observations relevant to the prevalence, healthcare costs and management of NCDs in PLHIV: (1) common NCDs in PLHIV were hypertension and diabetes mellitus, (2) consistently higher NCD prevalence rates in PLHIV compared with HIV-negative people, (3) consistently higher NCD prevalence rates from studies conducted outside Africa, with fewer studies and inconsistent NCD prevalence rates reported from SSA studies, (4) fewer longitudinal case–control and interventional studies to determine NCD prevalence and related determinants (5) integrated management of HIV–NCD co-morbidity might be more cost-effective than vertical HIV and NCD programmes and (6) fewer studies reporting the capacity of ART clinics to manage NCDs in PLHIV.

The review utilised data from several study contexts. In most situations, key variables such as gender, age groupings and different co-morbid diseases were categorised differently, as can be observed from the presented findings. Again, commonly known key variables like race, HIV subtypes and population groups (e.g. heterosexuals and men having sex with men [MSM]) were not explicitly stated in the studies; and therefore their influence on study findings could not be assessed.

Hypertension was the most common form of CVDs assessed in the studies reviewed, where the prevalence of hypertension was found to be consistently higher in PLHIV than in HIV-negative controls. Marked variations in prevalence of hypertension were noted between studies conducted outside Africa (Asia, America and Europe) and those conducted in Africa (Zimbabwe and Malawi). Higher rates of hypertension were observed in studies conducted in Asia, Europe and America. Most (3) of the studies in Africa were conducted in Zimbabwe, where the prevalence rates were lower than those conducted outside Africa, and varied widely from 10% to 30%.^9,12,13^ Diabetes mellitus was an often-mentioned metabolic condition in studies on the prevalence of NCDs in PLHIV, where the prevalence of diabetes mellitus was again found to be consistently higher in PLHIV than in HIV-negative controls. The prevalence rates varied widely from 1% in Zimbabwe to 26% in Japan. However, studies conducted outside Africa (in Asia, America and Europe) also found higher rates of diabetes mellitus in PLHIV compared with those conducted in Africa. Few (3) studies conducted in Zimbabwe showed marked variation in the prevalence of diabetes mellitus, ranging from 1.0% to 8.3%. Respiratory diseases (only asthma in Zimbabwe) and cancers were the least reported NCDs and the least prevalent in PLHIV. With regard to cancers, Haregu et al.^22^ point out that in the era of ART, both AIDS-defining and non-AIDS-defining cancers have become very low in PLHIV to almost resemble the prevalence in HIV-negative people.^22^

Most of the studies (15 studies) used cross-sectional designs, and the remaining ones used retrospective (four studies) and modelling (model assumptions) designs (two studies) to determine the prevalence of NCDs. Determining the prevalence rate using cross-sectional studies through point-of-care screening might not be reliable and repeat visits might be needed to confirm diagnoses, whilst using the existing records might be prone to missing/incomplete data and various forms of errors. None of the prevalence rates for diabetes mellitus and hypertension was determined through longitudinal studies to provide stronger evidence on prevalence.

One study conducted in Africa showed evidence of limited capacity of ART sites to screen and treat NCDs in PLHIV.^21^ In Africa, the inconsistencies in the prevalence rates of hypertension and diabetes mellitus might be explained in part by the limited capacity of ART centres to screen and/or diagnose hypertension and diabetes mellitus because of various health system and patient-level challenges. This limited capacity could result in missed opportunities for screening and diagnosis and consequently lower prevalence being recorded as observed on African studies.^21^ Challenges to ensure follow-up diagnostic tests and patients’ attendance to screening appointments were often noted in studies reviewed.^21,23^ Besides the few studies assessing the prevalence of hypertension and diabetes in both the general population and in PLHIV at country level, there are no published studies assessing the capacity of ART centres to screen for hypertension and diabetes mellitus and the associated challenges in African studies. Again, no study was found that assessed how NCDs in PLHIV are being managed and the existing challenges from both the perspective of the healthcare providers and that of the clients.

Key findings point to a general increase in NCDs amongst PLHIV. The increase in NCD incidence, in addition to the commonly known risk factors, seems to be associated with the length of duration of HIV infection and the duration on ART.^7,8,13^ Commonly identified NCDs in PLHIV were CVDs (especially hypertension) and diabetes mellitus, with evidence suggesting the incidence of these NCDs is likely to increase further in the near future.^9,10^ Besides the consistent findings on hypertension and diabetes mellitus being more prevalent in PLHIV, no studies assessed the access to NCD treatment in PLHIV and how the access is influenced by the key determinants of health (income, employment status, distance to health facilities and number of co-morbidities). Circumstances for PLHIV alone and for those living with co-morbid HIV and NCDs may differ by context, as defined by various social determinants of health at individual, community and country levels. These determinants of health may in turn shape the health outcomes for PLHIV differently according to health status, context and individual circumstances.

Managing HIV–NCD co-morbidity was generally found to be more cost-effective or cost saving compared with managing HIV or NCDs separately. More efficient resource utilisation may be achieved by integrated management of HIV and NCDs.^18,19^ NCD medication and laboratory services were found to be major cost contributors. Integrated HIV–NCD screening was generally found to result in reduced overhead costs, patient transport costs and time burden, from societal perspective. More implementation studies are required that will assess the cost-effectiveness of integrating the various types of key NCDs in the unique context of Africa, where the implementation of integrated HIV–NCD care is not yet fully in place.

Available evidence from high-income countries (HICs) did not find a statistically significant impact of co-morbidities on healthcare costs (out-of-pocket expenses) amongst PLHIV who were commercially insured.^10,19^ However, the review could not find similar studies conducted on LMICs such as Zambia, Mozambique and Zimbabwe. Health financing for countries at different levels of development may vary significantly, thereby differentially affecting the impact of co-morbidities on healthcare costs in PLHIV. In most LMICs like Zimbabwe, the greater proportion of people do not have health insurance, and health financing is largely out of pocket.^24^ Ability to pay becomes a major determinant of healthcare access in people without health insurance, which is likely to produce inequities in health outcomes based on the ability to pay.

### Study limitations

Search outputs were limited to those written in English language, thereby missing out relevant literature published in other commonly used languages like French and Chinese and potentially impacting negatively on the generalisations made in this review.

There were also differences in study contexts, and the potential for variation, in study population characteristics compared. The studies compared materials from different gender and age groupings and different co-morbid diseases. In all the studies reviewed, distinctions on key study population characteristics were not reported such as race, HIV subtypes (e.g. subtype B vs. C) and population groups based on sexual orientation (e.g. heterosexuals and MSM). This could have negatively affected the findings and interpretations made from the study.

## Conclusion

Results from this review show higher prevalence rates of diabetes mellitus and hypertension in PLHIV compared with HIV-negative people. However, the findings show inconsistency in NCD prevalence data from studies conducted in SSA countries and also limited research evidence on the capacity of ART sites to manage NCDs in PLHIV. Low prevalence rates of NCDs reported on SSA could be an indication of limited capacity to screen for NCDs as a result of the influence of health-system and/or patient-level factors. Most studies conducted on prevalence and healthcare costs in PLHIV are generally limited to cross-sectional studies, with very few interventional, longitudinal studies.

## Recommendations

Studies that will assess the influence of co-morbid HIV–NCDs in PLHIV, the cost-effectiveness of integrating NCD care with HIV care and the capacity of ART sites to screen and manage NCDs are recommended to inform future interventions.

Furthermore, longitudinal case–control and intervention studies are recommended for more accurate determination of NCD incidence and prevalence data, both in PLHIV and in HIV-negative population.

It might also be worthwhile if future studies can assess the independent role played by various population characteristics (like age, race and HIV sub-types) in explaining the NCD prevalence and healthcare costs for PLHIV.
